# Antibiotic Resistance and Molecular Profiling of the Clinical Isolates of *Staphylococcus aureus* Causing Bovine Mastitis from India

**DOI:** 10.3390/microorganisms10040833

**Published:** 2022-04-18

**Authors:** Umarani Brahma, Akash Suresh, Shweta Murthy, Vasundhra Bhandari, Paresh Sharma

**Affiliations:** 1 Department of Infectious Diseases, National Institute of Animal Biotechnology, Hyderabad 500032, India; umarani.brahma@gmail.com (U.B.); akash.hawkz@gmail.com (A.S.); shwetanori@gmail.com (S.M.); 2 Manipal Academy of Higher Education, Manipal 576104, India; 3 Department of Pharmacoinformatics, National Institute of Pharmaceutical Education and Research (NIPER), Hyderabad 500037, India

**Keywords:** bovine mastitis, MRSA, biofilm, antimicrobial susceptibility, multidrug-resistance, virulence

## Abstract

*Staphylococcus aureus* is an opportunistic bacterium known to cause severe infections in humans and animals. It is one of the major bacteria causing subclinical and clinical mastitis, leading to significant economic losses in livestock industry. In this study, we have isolated and characterized 80 *S. aureus* clinical isolates from mastitis-infected animals. The analysis of antimicrobial susceptibility, molecular typing, biofilm production and genetic determinants was performed to understand molecular and phenotypic features of the prevalent pathogen. Our antibiotic susceptibility assays showed the majority (57.5%) of isolates to be multidrug-resistant (MDR), 38.75% resistant and 3.75% sensitive. We found 25% isolates to be methicillin-resistant *S. aureus* (MRSA) based on oxacillin susceptibility assays. In the MRSA group, maximum isolates (95%) were MDR compared to 45% in MSSA. Multilocus sequence typing (MLST) revealed 15 different STs; ST-97 was the most common ST, followed by ST-2459, ST-1, ST-9 and ST-72. The *agr* typing showed *agr*-I as the most common type, followed by type II and III. Most isolates developed biofilms, which ranged in intensity from strong to weak. The presence or absence of lukS, a virulence-related gene, was found to have a substantial relationship with the biofilm phenotype. However, no significant association was found between biofilm formation and antimicrobial resistance or other virulence genes. We also found four MRSA isolates that were *mecA* negative based on molecular assays. Our findings reveal the prevalence of multidrug-resistant *S. aureus* clinical isolates in India that are biofilm positive and have critical genetic factors for disease pathogenesis causing bovine mastitis. This study emphasizes the need for the comprehensive surveillance of *S. aureus* and other mastitis-causing pathogens to control the disease effectively.

## 1. Introduction

The emergence of antibiotic-resistant pathogens in livestock poses a significant threat to animal and human health. *Staphylococcus aureus* is a deadly pathogen that affects a broad host range causing mild to severe infections [1,2]. In animals, it is mainly responsible for a subclinical and clinical form of bovine mastitis [3]. In subclinical mastitis, symptoms are not visible; however, in clinical mastitis, milk yield decreases and milk color changes are observed. Infections caused by *S. aureus* are generally contagious and difficult to treat with antibiotics, especially in the case of methicillin-resistant *S. aureus* (MRSA) [3,4]. MRSA isolates are resistant to β-lactam antibiotics as they harbor the *mecA* gene, which renders the drug inactive, resulting in treatment failure. They also show an oxacillin minimum inhibitory concentration of ≥4 µg/mL and are frequently resistant to other antibiotics [2,5]. There are several reports of MRSA causing bovine mastitis across the globe with the potential for zoonotic transmission [6,7]. Infection due to MRSA results in increased antibiotic use and is generally associated with multidrug resistance [5]. Another concerning factor associated with *S. aureus* is their ability to form biofilms, which safeguard them from the host immune system and antibiotics effect. The pathogenic isolates with biofilm production represent a more dangerous threat [8,9]. They are also known to release numerous toxin and virulence factors, which cause food poisoning toxic shock syndrome and help in disease pathogenesis [1,4,10]. Until now, a wide lineage of *S. aureus* clones has been identified that circulate in different regions and countries and exhibit different antimicrobial, virulence and molecular characteristics [5,11,12]. However, there are limited data on the clones of *S. aureus* and their virulence profile causing bovine mastitis, mainly in India.

To formulate an appropriate treatment regimen and control measures for bovine mastitis, studies on the molecular and phenotypic characteristics and epidemiology of *S. aureus* infections are essential. In this study, we aimed to characterize the antimicrobial, moleulcar type, virulence and biofilm profile of the *S. aureus* bovine mastitis isolates currently prevalent in India. 

## 2. Materials and Methods

### 2.1. Sample Collection and Isolation of Bacteria

Milk samples were collected from cows with clinical mastitis showing symptoms of udder inflammation, reduced milk yield, clots and changed milk color. Milk samples were collected from different states of India, Telangana (*n* = 155, 6 farms) Andhra Pradesh (*n* = 90, 5 farms) and Tamil Nadu (*n* = 188, 6 farms). Milk samples were collected from infected teats of each animal. Before collection, the teats were cleaned, followed by the disposal of first few drops of milk and then again cleaned with 70% ethanol. The sample was added into trypticase soy broth (TSB) (Himedia, Mumbai, India) and incubated at 37 °C for 16 h. The broth with visible bacterial growth was then streaked on mannitol salt agar (MSA) (Himedia, Mumbai, India) plates and incubated at 37 °C for 16–20 h. Colonies with yellow-colored zones were assumed to be *S. aureus*.

### 2.2. Biochemical and Molecular Characterization of S. aureus Isolates

The yellow colonies were further confirmed by biochemical tests such as Gram staining (Sigma Aldrich, Stenheim, Switzerland), catalase assay (BBL™ BD catalase reagent droppers, Maryland, USA) and latex agglutination test (detects the protein A and clumping factor, (HiStaph™ Latex Test Kit, Himedia, Mumbai, India)) and coagulase test (Himedia, Mumbai, India). Furthermore, 16S rRNA gene sequence analysis was performed to reconfirm the bacterial species. Genomic DNA was extracted with slight modifications using the Wizard genomic kit (Promega, Madison, WI, USA). Briefly, 5 mL of the culture was pelleted down at 3000× *g* for 10 min. The pellet was washed twice with 1XPBS and suspended in 500 µL of 50 mM EDTA containing 100 µg/mL lysozyme for 2 h at 37 °C before following the manufacturer’s protocol. DNA quality was assessed using Nanodrop (Thermo Scientific, Waltham, MA, USA) and diluted using sterile deionized water. The 16S rRNA gene was amplified as described previously [13], and the product was sequenced by Sanger sequencing at Bioserve Pvt Ltd., Secunderabad, India.

### 2.3. Multilocus Sequence Typing (MLST)

MLST typing was performed by amplifying the internal fragments of 7 housekeeping genes (*2rc*, *aroE*, *glpF*, *gmk*, *pta*, *tpi* and *yqil*) using the protocol described in http://www.mLst.net., accessed on 15 May 2017. The amplified PCR products were then sequenced using the Sanger sequencing method at Bioserve India Pvt Ltd. Each isolate’s sequence type (ST) was determined by comparing *S. aureus* strains sequences within the available MLST database.

### 2.4. Accessory Gene Regulator (agr) Typing

The *agr* typing of each isolate was performed by multiplex PCR (Bio-Rad, Hercules, CA, USA) using four primers *pan*-F (5′-ATGCACATGGTGCACATGC-3′); *agr*1 (5′-GTCACAAGTACTATAAGCTGCGAT-3′); *agr*2 (5′-TATTACTAATTGAAAAGTGGCCATAGC-3′); *agr*3 (5′-GTAATGTAATAGCTTGTATAATAATACCCAG-3′); and *agr* 4 (5′-CGATAATGCCGTAATACCCG-3′), as described earlier [14]. The amplification conditions followed were as follows: 94 °C for 5 min followed by 26 cycles of 94 °C for 30 s, 55 °C for 30 s and 72 °C for 60 s and a final cycle of 72 °C for 10 min. The amplification of variable product sizes was observed for *agr* type I (441 bp), *agr* type II (575 bp), *agr* type III (323 bp) and *agr* type IV (659 bp).

### 2.5. Antibiotic Susceptibility Assay

Susceptibility assays were performed using disk diffusion and microbroth dilution methods. All antibiotics discs were purchased from (Himedia, Mumbai, India). ATCC 29213, ATCC 700699 and ATCC 25923 were used as internal controls as per CLSI guidelines [15]. The microbroth dilution method was used to determine the MIC’s of oxacillin, vancomycin and linezolid (Sigma, Bangalore, India) (0.0–32 µg/mL) using resazurin (Sigma, Bangalore, India) dye as described earlier [16]. Disk diffusion assay was performed for cefoxitin (30 µg), clindamycin (2 µg), erythromycin (15 µg), gentamicin (10 µg), rifampicin (5 µg), teicoplanin (30 µg) and tetracycline (30 µg). Strains with resistance against three or more classes of antibiotics were considered multidrug-resistant (MDR), against one or two antibiotics considered resistant (R), and strains susceptible to all classes of antibiotics were described as sensitive (S) in the manuscript.

### 2.6. Biofilm Formation

Biofilm forming capacity was assessed using the crystal violet (CV) (Sigma, Bangalore, India) method, as described earlier with slight modifications [17,18]. In brief, 200 µL of overnight grown culture (1:200 dilution) was suspended to each well of a 96-well plate and incubated for 24 h at 37 °C. After 24 h of incubation, the plates were washed thrice with 1 × PBS followed by 15 min incubation with 200 µL of methanol. The plates were air-dried for 20 min, and 0.2% crystal violet (100 µL) was added and incubated at room temperature for 15 min. After incubation, plates were washed with distilled water, and 100 µL of 33% acetic acid was added to dissolve the formed biofilm. Biofilm formation was measured at an absorbance of 590 nm using a multimode reader (Perkin Elmer, New Jersey, NJ, USA). The intensity of biofilm formation was calculated using the below method described earlier [17].

O.Dc ≥ O.Ds => non-adherent. O.Dc < O.Ds ≤ 2 × O.Dc => Weak. 2 × O.Dc < O.Ds ≤ 4 × O.Dc => Moderate. 4 × O.Dc < O.Ds => Strong

[O.Dc = Optical Density of control; O.Ds = Optical Density of sample]

### 2.7. Virulence and Antibiotic Resistance Determinants Gene Profiling

PCR (Bio-Rad, Hercules, CA, USA) was performed to check for genes involved in antibiotic resistance, virulence, adhesion, biofilm formation and pathogenesis in all isolates. Antibiotic resistance genes included in the study were methicillin (*mecA* and *mecC*) and vancomycin resistance determinants (*vanA*); virulence and toxin genes studied were leukocidin genes (*lukMF*, *lukS* and *lukF*), haemolysin genes (*α-hls*, *β-hls*, *γ-hls* and *δ-hls*), biofilm formation and adhesion genes (*icaA*, *icaD*, *bap*, *clfA*, *clfB*, *fnbA* and *fnbB*) and genes involved in pathogenesis (*cna*, *adsA*, *sbi* and *scn*). The primers were designed from *S. aureus* specific gene sequence using NCBI- Primer Blast software. The primer sequence and PCR information are mentioned in Table 1.

### 2.8. eBUSRT Analysis

The relationship between ST and virulence gene or antimicrobial resistance profile was analazed using eBURST analysis. The minimum spanning tree was constructed by the goeBURST algorithm using Phyloviz software v1.1 [19].

### 2.9. Statistical Analysis

χ^2^ and ANOVA tests were performed using GraphPad PRISM 7.00. We used the two tests to identify statistical significance and correlation between antimicrobial resistance and biofilm formation or antimicrobial resistance and virulence genes across all 80 *S. aureus* strains. *p*-value < 0.05 was considered statistically significant.

## 3. Results

### 3.1. Antimicrobial Profiling of Clinical Isolates

A total of 433 milk samples were collected from mastitis infected animals, from which 80 *S. aureus* clinical isolates were obtained. Antibiotic susceptibly screening of these isolates was performed against some of the commonly used drugs in the field. It showed a maximum resistance against gentamicin (58.75%, 47/80) followed by clindamycin (53.75%, 43/80), erythromycin (40%, 32/80), rifampicin (35%, 28/80), tetracycline (30%, 24/80), oxacillin (25%, 20/80), cefoxitin (25%, 20/80) and teicoplanin (22.50%, 18/80) (Table 2). No resistance was detected against vancomycin and linezolid, although a few isolates showed the borderline sensitivity of 2 µg/mL against vancomycin in the microbroth dilution assay.

Twenty-five percent of the isolates were characterized as MRSA based on its resistance to Oxacillin. To further understand the resistance profile difference between the MRSA and MSSA isolates, we annotated isolates showing resistance against one or two drugs as resistant and more than two drugs as MDR. Our results revealed the majority of MRSA isolates (*n* = 19) as MDR, whereas one was resistant (Table 2). On the other hand, in the MSSA category, 27 out of 60 were MDR, 30 were resistant and 3 isolates showed the sensitive phenotype (Table 2).

### 3.2. Antibiotic Resistance Gene and SCCmec Typing

*S*. *aureus* is known to harbor various antimicrobial resistance genes, and some of the commonly used genes for detecting resistant *S. aureus* variants are *mecA*, *mecC* and *vanA*. We performed PCR-based screening for the presence of these genes across isolates. Sixteen isolates were positive for the *mecA* gene; however, none of the isolates were positive for *mecC* and *vanA* genes (Table 3). To understand the diversity of *mecA* gene, SCC*mec* typing of the *mecA* positive isolates was performed. It was observed that Type III (*n* = 7) was the most prevalent SCC*mec* type across the isolates followed by Type II (*n* = 4), Type IVa (*n* = 4) and Type IVd (*n* = 1) (Table 3).

### 3.3. Molecular Typing of S. aureus Isolates

**Figure 1:**
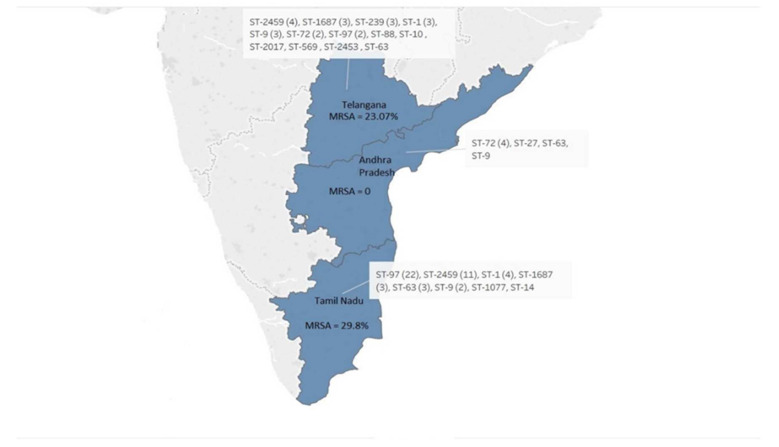
Distribution of sequence types (ST) of 80 clinical isolates of *S. aureus* and prevalence of MRSA among three different states: Telangana, Andhra Pradesh and Tamil Nadu. ST was determined using the multilocus sequence typing (MLST) method.

MLST and *agr* typing analysis was performed to understand genetic diversity across our isolates. MLST analysis of the 80 isolates revealed high genetic diversity among them. A total of 15 different STs were identified, of which ST-97 (*n* = 24) was the most common. ST- 2459 (*n* = 15), ST-1 (*n* = 8), ST-9 (*n* = 6), ST-72 (*n* = 6), ST-1687 (*n* = 5), ST-63 (*n* = 5), ST-239 (*n* = 3) and isolates each representing ST-10, ST-14, ST-27, ST-88, ST-569, ST-1077, ST-2017 and ST-2453 were also found (Figure 1). The sequence types ST-9 and ST-63 were common among the three states.

**Figure 2:**
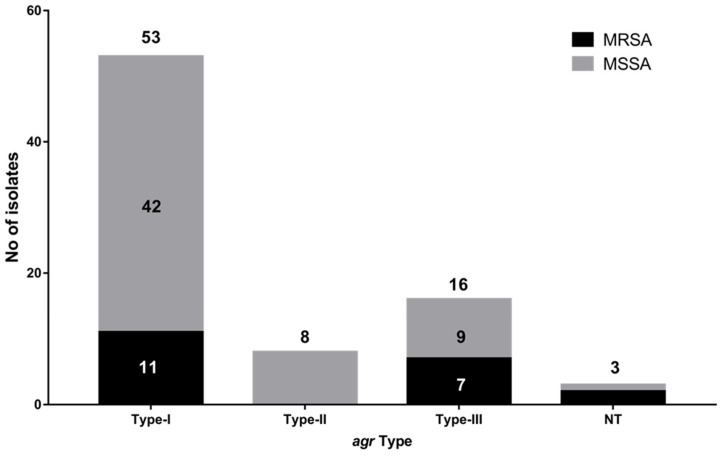
Molecular characterization of the *S. aureus* clinical isolates using accessory gene regulator locus (*agr*) typing: *agr* Type-I was the most common among the isolates followed by type III. Type-II was not found in the MRSA isolates but there were 2 and 1 NTs in MRSA and MSSA, respectively.

*agr* typing revealed type I as predominant in 67% of isolates followed by type III (20%) and II (10%) (Figure 2). However, three isolates were found to be Non-Typeable (NT). Fifty-five percent of MRSA isolates belonged to type I, followed by 35% in type III and 10% non-typeable isolates. In case of the MSSA group, the majority of the isolates belong to type I (71.1%), followed by type III (15.2%) and II (13.5%) with one non-typeable isolate.

### 3.4. Biofilm Production, Virulence and Toxin Genes Profiling

**Figure 3:**
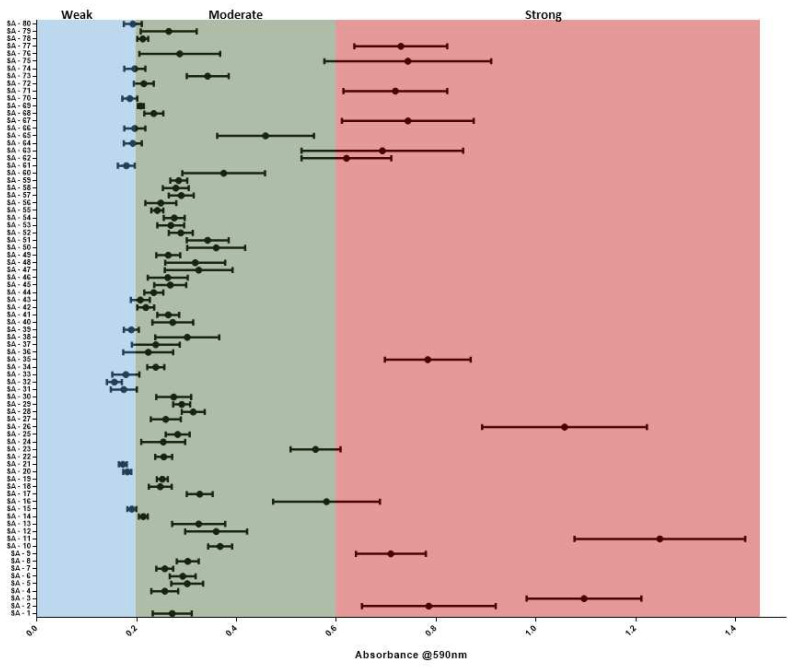
Classification of the biofilm-forming intensity of *S. aureus* isolates: The biofilm formation ability was determined using the crystal violet (CV) method and the biofilm formed was differentiated into strong (red), moderate (green) and weak (blue) biofilms.

**Figure 4:**
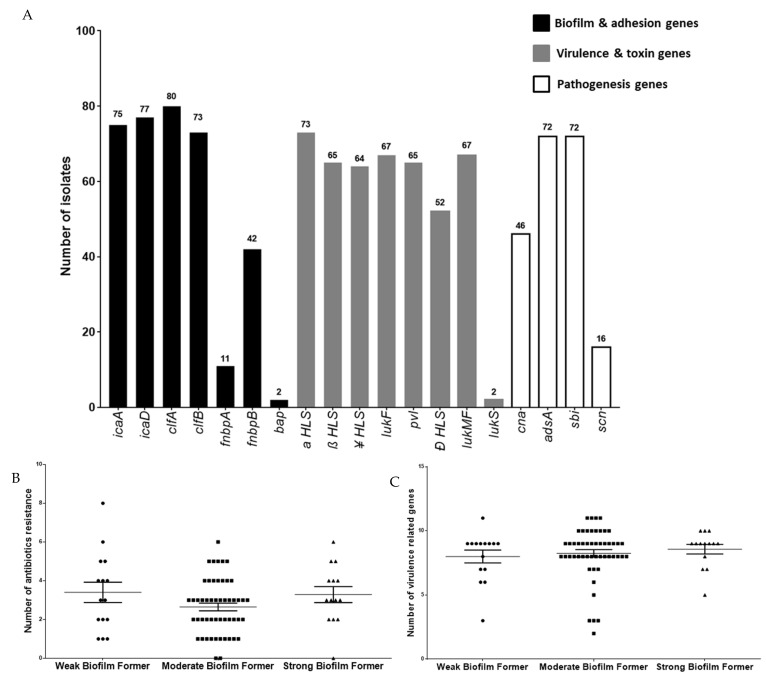
Genetic determinants profiling of markers: PCR-based detection of genes associated with biofilm formation, adhesion, virulence, toxin production and pathogenesis across *S. aureus* isolates (*n* = 80). (**A**) Biofilm and adhesion genes (*icaA*, *icaD*, *clfA*, *fnbpA*, *fnbpB* and *bap*), virulence and toxin genes (*aHLS*, *bHLS*, *cHLS*, *dHLS*, *lukF*, *pvl*, *lukMF* and *lukS*), pathogenesis genes (*can*, *adsA*, *sbi* and *scn*). (**B**) Comparison of antibiotic resistance in each isolate with respect to their biofilm formation. (**C**) Comparison of virulence genes in each isolate with respect to their biofilm formation.

Biofilm formation ability of *S. aureus* helps in its survival against antibiotics and host defenses, making biofilm formation a major concerning property of *S. aureus*. Biofilm-forming capacity was assessed in all isolates, which revealed moderate biofilm (*n* = 51) formation in majority of isolates, followed by weak (*n* = 15) and strong (*n* = 14) biofilm forming isolates (Figure 3). Next, the isolates were checked for the presence of various biofilm formation and adherence genes, *clfA* was prevalent in all, whereas *clfB* was present in 91.25% of the isolates. Fifty-two point five percent (52.5%) of the isolates showed *fnbB*, while *fnbA* was found only in 86.25%, (69/80) (Figure 4A). Biofilm producing genes such as *icaD* (96.25%, 77/80) and *icaA* (93.75%, 75/80) were also present while, *bap* gene was found in only two isolates (Figure 4A). Further virulent and toxin genes were also checked in the isolates, and *α-hls* gene was predominant and present in 91.25% of the population, followed by 83.75% of *lukMF* and *lukF*, 81.25% of *pvl* and *β-hls*, 80% of *γ-hls* and 65% of *δ-hls;* however, only 2.5% isolates had the *lukS* gene (Figure 4A). In genes related to pathogenesis, 90% of the isolates contain *adsA* and *sbi*, followed by 57.5% *cna* and 20% of *scn*.

Statistical analysis using ANOVA showed that there is no significant change in the number of antimicrobial the isolate is resistant against with changes in the biofilm type. The same result was obtained in the case of the number of virulence associated genes and biofilm type (Figure 4B,C).

### 3.5. Significance of Presence and Absence of Gene across S. aureus Isolates

**Figure 5:**
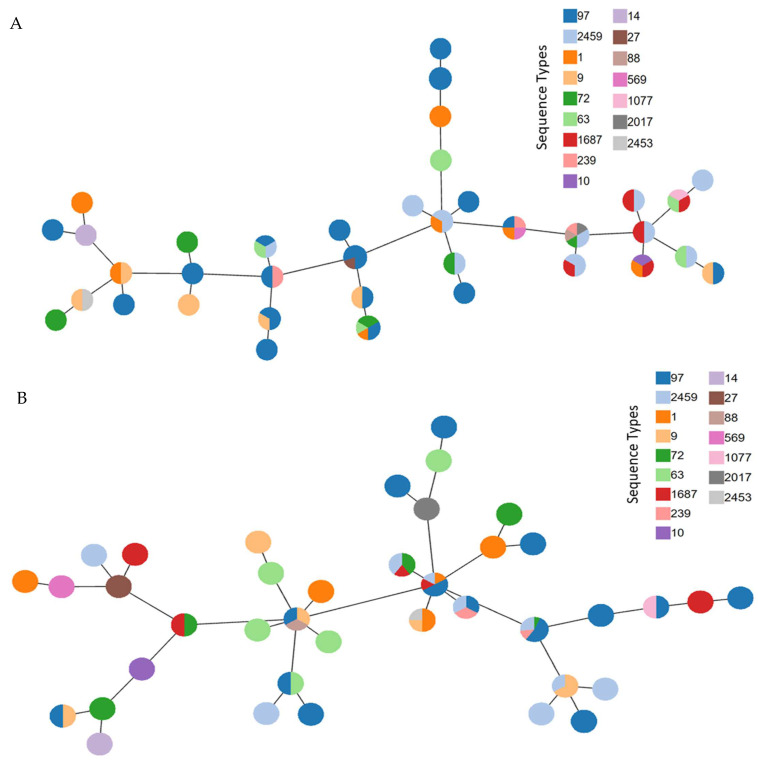

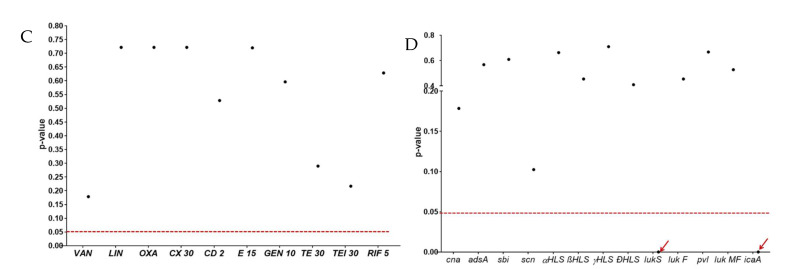
Significance of virulence genes and antimicrobial resistance across *S. aureus* isolates (*n* = 80). (**A**) eBURST analysis showing the random distribution of antimicrobial resistance profile across different MLST. (**B**) eBURST analysis showing the random distribution of virulence genes across different MLST. (**C**) Chi-square analysis of antimicrobial resistance and susceptibility with respect to biofilm formation. (**D**) Chi-square analysis of virulence genes with respect to biofilm formation. The red-dotted line marks the cutoff value of 0.05, the values falling below the cutoff range are considered statistically significant and the red arrows mark the genes with a significant *p*-value (<0.05).

eBURST analysis was performed to identify the distribution of antimicrobial resistance and virulence genes across different MLST types of *S. aureus* isolates (Figure 5A,B). The nodes were created on the basis of the resistance or sensitive phenotype against the screened set of antimicrobials (mentioned in Section 3.1) (Figure 5A). It was observed that the antimicrobial resistance profile of strains was distributed across different ST types among the isolates used in this study. The same outcome was achieved when the nodes were formed on the basis of the presence or absence of selected virulence-associated genes (mentioned in Section 3.4) (Figure 5B). The result in Section 3.5 had shown that there is variability in biofilm formation across all isolates. Since biofilm formation has shown to be influenced by both antimicrobial resistance profile and virulence of an organism [20,21], we performed a chi-square test of various aforementioned virulence genes and AMR profiles of strains used in this study against biofilm formation (Figure 5C,D). Even though none of the antimicrobial resistance profile have shown significant impacts on biofilm formation as per chi-square test; but in case of virulence genes, it was the opposite. It was observed that *lukS* showed statistically significant impact on biofilm formation among the isolates, as its *p*-value was below the 0.05 cutoff along with *icaA*, a biofilm formation gene that was taken as a positive control for the study (Figure 5D).

## 4. Discussion

Bovine mastitis caused by *S. aureus* is a huge concern globally due to its multiplex nature, which makes its treatment and control difficult. Hence, it is vital to understand these pathogenic bacteria’s overall characteristics and lineages before devising any control or elimination policy. From India, there is a paucity of data on the molecular characterization and lineages of these pathogenic isolates causing mastitis. Therefore, in the present study, we have isolated *S. aureus* clinical strains from cattle’s suffering from mastitis and characterized them based on various molecular and phenotypic properties. Some of the highly concerning attributes of bovine mastitis are multidrug resistance, biofilm formation and high virulence. 

The antimicrobial profiling of these isolates against 10 antibiotics revealed 57.5% of the isolates as MDR, which is similar to other studies from India (62%), China (64.8%) [5] and Bangladesh (49%) [2,3,12]. Furthermore, the characterization was performed based on cefoxitin and oxacillin susceptibility, which showed 25% isolates to be MRSA out of which 80% were *mecA* positive. The remaining 20% MRSA isolates were *mecA* negative and might have an alternative mechanism for methicillin resistant phenotype. Other reports from India and different regions also showed a similar prevalence of MRSA, ranging from 14.3 to 29% [3,22,23,24]. Reports of *mecA* negative MRSA have been published earlier from human and animal infections [25,26]. Among the MRSA isolates, 95% were MDR, while in the MSSA group, 45% were MDR. The prevalence of high drug resistance across isolates showed the misuse of antimicrobials in the field. A change in the approach used in the treatment or improving the regulation involving unnecessary antibiotic usage in the farms can help reduce AMR emergence.

Molecular typing of the isolates based on MLST, *agr* tying and SCC*mec* typing revealed a heterogeneous population. The large-scale transport of cattle across different region can be one of the major causes for the development of such mixed population of isolates across different regions. Comparison of MLST data from three states revealed *S. aureus* isolates from Telangana to be most diverse; however, ST-9 and ST-63 were the only common sequence types among the three states. Additionally, 30% of the isolates belonged to ST-97, which is in line with reports from China where ST-97 is the dominant sequence type causing mastitis [5,12]. The majority of the isolates (66.25%) belonged to *agr* I type, which is similar to earlier studies from India (87.3%, 151/173 and 66.7%, 26/39 [2,27], indicating that this group predominantly caused mastitis while compared with other *agr* group strains [11]. Correlation between ST and *agr* type divulged that ST-2459, ST-239, ST-2017 and ST-569 all were *agr* type I isolates. In addition to that, 20 isolates with ST-97 also belonged to *agr* type I. However, no association of MRSA or MSSA isolates with ST or *agr* types could be seen, indicating random distribution. eBURST analysis also showed that the distributions of antimicrobial resistance profile and virulence genes are independent of the ST types among the isolates.

In addition to their antibiotic resistance phenotype, biofilm production ability was also analyzed, which provides them with an extra advantage for survival in the host. The bacterial community within the biofilm has reduced exposure to antibiotics and host immune attack [28], resulting in a contribution both in antimicrobial resistance and virulence of a pathogen [20,21]. Hence, varying biofilm formations across *S. aureus* isolates were expected to be affected by either antimicrobial resistance or virulence genes. However, resistance against the number of antibiotics or the presence of a number of virulence genes did not correlate with biofilm phenotype. The majority of the MRSA isolates (55%) formed moderate biofilms, followed by weak (25%) and strong (20%) biofilms. A similar observation was seen in the case of MSSA isolates with 66.6% moderate biofilms followed by 16.6% of weak and strong biofilms each. Such varying biofilm formation levels led us to investigate if this is due to the varying numbers of virulence genes or resistance against antimicrobials. However, the ANOVA test (*p* > 0.05, non-significant) revealed that both factors were independent of biofilm formation. Furthermore, the correlation between the presence or absence of virulence genes on biofilm formation showed *lukS* absence to impact biofilm formation. Biofilm formation requires intracellular adhesion gene cluster *icaADBC* and genes *icaA* and *icaD*. Out of the 80 isolates studied, *icaA* and *icaD* were detected in 93.75% and 96.25% of the isolates, respectively. A similar prevalence of these genes was reported from countries such as Belgium, Rio de Janerio and China [29,30,31]. The *bap* gene, which is implicated in biofilm formation in S. aureus isolates from mammary infections [32], was found in two isolates, which were similar to previous studies from India, where only one isolate [9] was reported positive, and a group from Spain [31] reported that 5% positive isolates. *S. aureus* secretes various toxins, which allows them to survive and attack hosts, and a few of these toxins secreted in the milk may result in food poisoning. The *pvl* gene responsible for the pore formation of host cells was detected in 81.25% of isolates. In previous reports from India, they were detected in 10.3 to 82 % of the isolates, whereas in other published reports, it was found in 50% isolates from Italy and 41.5% in China, [2,5,33,34,35]. It contrasts with other studies where no *pvl* positive isolates were found [1,23,25,33]. Eighty percent of the MRSA isolates were *pvl* positive, which further increases the chance of chronic infections. The exotoxins such as *α-hls*, (91.25%), *β-hls* (81.25%), *γ-hls* (80%) and *δ*-*hls* (65%) were also detected in the majority of the isolates, which is similar to the previous report where all isolates were positive for *α*-*hls* and *δ*-*hls*, while *β-hls* and *γ-hls* were present, respectively, in 86% and 75% [30]. Another study from China reported that more than 80% of the isolates were positive for *α-hls*, *β-hls* and *δ*-*hls* [4,30]. 

Another fascinating trait of *S. aureus* is its capacity to cling to cell surfaces and colonise them in order to survive and spread. The current investigation discovered *fnbB* (52.5%) and *fnbA* (86.25%) genes in isolates, both of which aid in cell adhesion; these findings were consistent with Soares et al., 2017 who detected 78.2 percent of *fnbB* and 27.3 percent of *fnbA* [30,36]. Similar investigations from China found a high prevalence of both genes in bovine mastitis *S. aureus* isolates [31]. The existence of these genes is the first step toward pathogenesis via fibronectin adherence [37]. Clumping factor genes *clfA* and *clfB* are among the adhesion genes that assist clump blood plasma and initiate infection [38]. The *clfA* gene was found in all isolates in our investigation, whereas *clfB* was positive in only 91.25% isolates. Earlier studies also reported 84% to 100% of the population harboring both genes [39,40]. Collagen-binding protein, or *cna*, is another factor in S. aureus pathogenicity. In this investigation, 57.5% of isolates tested positive for *cna*. Few other studies report a varying percentage from 22.4% to 65.3% [10,39,41,42]. However, a report by Ahangari et al. states that this gene does not play an essential role in mastitis pathogenesis [39]. Staphylococcal complement inhibitor protein-coding gene *scn* was found in 20% of the isolates. In a study from South Africa, they could not identify the *scn* gene in any of the isolates [36]. Immunoglobulin binding protein, *sbi* and adenosine synthase A, *adsA*, have a predominant role in pathogenesis, and we found 90% of isolates to be positive. 

## 5. Conclusions

In this paper, we discovered that *S. aureus* isolates that cause mastitis in India constitute a diverse population with distinct lineages and molecular types. In addition to their variable antibiotic resistance phenotype, they also harbor various virulence and toxin genes enabling them to cause chronic infections in dairy animals. In the absence of a virulence-associated gene, *lukS* certain isolates displayed more robust biofilm formation and showed a statistically significant association between the two (*p*-value 0.001 upon chi-square test). Further research incorporating a larger number of samples from different geographical locations of the country in the future would provide a more comprehensive picture of the epidemiology of *S. aureus* isolates in India. The development of more sophisticated diagnostic methods and treatment options will be aided by a better understanding of the incidence of *S. aureus* in the Indian environment.

## Figures and Tables

**Table 1:** List of primers used in the study.

No	Primer	Sequence (5′-3′)	Size (Base Pairs)	Annealing Temperature (°C)
1	*bap* For	CCCTATATCGAAGGTGTAGAATTGCAC	971	55
2	*bap* Rev	GCTGTTGAAGTTAATACTGTACCTGC		
3	*clfA* For	ATTGGCGTGGCTTCAGTGCT	292	52
4	*clfA* Rev	CGTTTCTTCCGTAGTTGCATTTG		
5	*clfB* For	ACATCAGTAATAGTAGGGGGCAAC	205	52
6	*clfB* Rev	TTCGCACTGTTTGTGTTTGCAC		
7	*fnbA* For	CATAAATTGGGAGCAGCATCA	127	52
8	*fnbA* Rev	ATCAGCAGCTGAATTCCCATT		
9	*fnbB* For	GTAACAGCTAATGGTCGAATTGATACT	524	52
10	*fnbB* Rev	CAAGTTCGATAGGAGTACTATGTTC		
11	*icaA* For	TATACCTTTCTTCGATGTCG	561	48
12	*icaA* Rev	CTTTCGTTATAACAGGCAAG		
13	*icaD* For	AAACGTAAGAGAGGTGG	381	47
14	*icaD* Rev	GGCAATATGATCAAGATAC		
15	*cna* For	TTACACCAGACGGAGCAACA	498	55
16	*cna* Rev	ATGACCCATAGCCTTGTGGA		
17	*adsA* For	GATGACGTTAGAACGCGTGA	499	55
18	*adsA* Rev	CTCTAGGGCCACCGAACATA		
19	*sbi* For	GTTGGGGCAGCAACAATTAC	497	55
20	*sbi* Rev	GCTGCTGATTTATCGTGTGG		
21	*scn* For	ACTTGCGGGAACTTTAGCAA	319	55
22	*scn* Rev	TTTTAGTGCTTCGTCAATTTCG		
23	*α-hls* For	GAAAATGGCATGCACAAAAA	485	55
24	*α-hls* Rev	CCATATACCGGGTTCCAAGA		
25	*β-hls* For	GTGCCAAAGCCGAATCTAAG	504	55
26	*β-hls* Rev	TTTTTCGATCATGTCCAGCA		
27	*δ-hls* For	TAATTAAGGAAGGAGTGATTTCAATG	100	55
28	*δ-hls* Rev	TTTTTAGTGAATTTGTTCACTGTGTC		
29	*¥-hls* For	AGAAGATATCGGCCAAGGTG	497	55
30	*¥-hls* Rev	CTTGACCATTCGGTGTAACG		
31	*lukMF* For	CAACTTTGTCGCTAGGTCTAA	730	48
32	*lukMF* Rev	CGCTCGTATCGCCTGAATCTT		
33	*luk F* For	TGCAGCTCAACATATCACACC	507	55
34	*luk F* Rev	GCTTCAACATCCCAACCAAT		
35	*luk S* For	TGAGGTGGCCTTTCCAATAC	492	55
36	*luk S* Rev	CATCCATATTTCTGCCATACG		

**Table 2:** Antimicrobial resistance profile of the *S. aureus* isolates.

FILE	Van MIC µg/mL	Lin MIC µg/mL	Oxa MIC µg/mL	VAN	LIN	OXA	CX 30	CD 2	E 15	GEN 10	TE 30	TEI 30	RIF 5	Res/Sus	MRSA/MSSA
SA-1	2	2	<0.5	S	S	S	S	R	S	S	R	S	S	R	MSSA
SA-2	2	2	<0.5	S	S	S	S	R	R	R	R	S	S	MDR	MSSA
SA-3	2	2	<0.5	S	S	S	S	R	R	R	R	S	S	MDR	MSSA
SA-4	2	2	<0.5	S	S	S	S	R	S	S	R	S	S	R	MSSA
SA-5	1	2	<0.5	S	S	S	S	R	S	S	S	S	S	R	MSSA
SA-6	2	2	0.5	S	S	S	S	R	S	R	S	S	R	MDR	MSSA
SA-7	1	2	0.5	S	S	S	S	R	S	S	S	S	S	R	MSSA
SA-8	0.5	4	<0.5	S	S	S	S	R	R	R	R	S	S	MDR	MSSA
SA-9	1	2	<0.5	S	S	S	S	R	R	R	R	S	R	MDR	MSSA
SA-10	2	2	1.25	S	S	S	S	S	R	S	S	S	S	R	MSSA
SA-11	1	2	<0.5	S	S	S	S	S	R	S	S	S	R	R	MSSA
SA-12	1	4	<0.5	S	S	S	S	S	S	S	S	S	S	S	MSSA
SA-13	1	2	<0.5	S	S	S	S	S	S	S	S	S	S	S	MSSA
SA-14	2	2	<0.5	S	S	S	S	S	R	R	R	S	S	MDR	MSSA
SA-15	2	2	<0.5	S	S	S	S	S	R	R	R	S	S	MDR	MSSA
SA-16	1	2	<0.5	S	S	S	S	S	S	S	S	S	S	S	MSSA
SA-17	1	2	<0.5	S	S	S	S	S	S	S	R	S	S	R	MSSA
SA-18	0.5	2	1.25	S	S	S	S	R	R	S	S	S	R	MDR	MSSA
SA-19	1	2	<0.5	S	S	S	S	R	R	S	S	S	R	MDR	MSSA
SA-20	1	2	0.63	S	S	S	S	S	R	S	R	S	S	R	MSSA
SA-21	1	2	0.63	S	S	S	S	R	R	S	R	S	S	MDR	MSSA
SA-22	1	2	0.63	S	S	S	S	S	R	S	R	S	S	R	MSSA
SA-23	2	4	8	S	S	R	R	S	S	S	R	S	S	MDR	MRSA
SA-24	1	2	8	S	S	R	R	S	S	R	S	S	R	MDR	MRSA
SA-25	1	2	0.5	S	S	R	R	S	S	R	R	S	S	MDR	MRSA
SA-26	1	4	0.5	S	S	S	S	S	S	R	S	R	S	R	MSSA
SA-27	1	2	0.5	S	S	R	R	S	S	R	R	S	S	MDR	MRSA
SA-28	1	4	0.5	S	S	R	R	S	S	R	R	R	S	MDR	MRSA
SA-29	1	4	0.5	S	S	R	R	S	S	R	R	S	S	MDR	MRSA
SA-30	1	4	0.25	S	S	S	S	S	S	R	S	S	S	R	MSSA
SA-31	2	4	0.25	S	S	S	S	S	S	R	S	S	S	R	MSSA
SA-32	1	4	0.25	S	S	S	S	S	S	S	S	R	S	R	MSSA
SA-33	2	4	0.25	S	S	S	S	S	S	R	S	R	R	MDR	MSSA
SA-34	1	2	0.13	S	S	S	S	S	S	R	S	R	S	R	MSSA
SA-35	2	4	0.5	S	S	S	S	S	S	R	S	R	R	MDR	MSSA
SA-36	1	4	0.25	S	S	S	S	S	S	R	S	S	S	R	MSSA
SA-37	1	4	0.25	S	S	S	S	R	S	R	S	R	S	MDR	MSSA
SA-38	2	4	8	S	S	R	R	S	S	R	S	R	R	MDR	MRSA
SA-39	1	4	0.5	S	S	S	S	R	S	R	R	R	S	MDR	MSSA
SA-40	1	2	0.25	S	S	S	S	R	S	R	S	R	S	MDR	MSSA
SA-41	1	2	0.5	S	S	S	S	R	S	R	S	S	R	MDR	MSSA
SA-42	1	4	0.5	S	S	S	S	R	S	R	S	S	S	R	MSSA
SA-43	1	2	0.5	S	S	S	S	S	S	R	S	S	S	R	MSSA
SA-44	2	2	8	S	S	R	R	R	S	R	S	S	S	MDR	MRSA
SA-45	1	2	0.25	S	S	S	S	R	S	R	S	R	S	MDR	MSSA
SA-46	1	2	8	S	S	R	R	R	S	R	S	R	S	MDR	MRSA
SA-47	1	4	0.5	S	S	S	S	S	S	R	S	R	R	MDR	MSSA
SA-48	2	2	0.5	S	S	S	S	S	S	R	S	R	R	MDR	MSSA
SA-49	1	4	0.5	S	S	S	S	S	S	R	S	R	R	MDR	MSSA
SA-50	1	2	0.5	S	S	S	S	S	S	R	S	S	S	R	MSSA
SA-51	1	4	8	S	S	R	R	R	R	R	R	R	R	MDR	MRSA
SA-52	1	2	<0.5	S	S	S	S	R	R	S	S	S	R	MDR	MSSA
SA-53	2	2	<0.5	S	S	S	S	R	R	S	R	S	R	MDR	MSSA
SA-54	0.5	2	<0.5	S	S	S	S	S	R	S	S	S	S	R	MSSA
SA-55	1	2	<0.5	S	S	S	S	R	R	S	S	S	S	R	MSSA
SA-56	0.5	2	<0.5	S	S	S	S	R	R	S	S	S	S	R	MSSA
SA-57	0.5	2	<0.5	S	S	S	S	R	R	S	S	S	S	R	MSSA
SA-58	1	2	<0.5	S	S	S	S	R	R	S	S	S	S	R	MSSA
SA-59	1	2	<0.5	S	S	S	S	R	S	S	S	S	S	R	MSSA
SA-60	2	2	0.5	S	S	S	S	R	R	S	S	S	S	R	MSSA
SA-61	1	2	<0.5	S	S	S	S	R	S	S	R	S	S	R	MSSA
SA-62	1	2	<0.5	S	S	S	S	R	R	S	R	S	R	MDR	MSSA
SA-63	0.5	2	<0.5	S	S	S	S	R	R	S	R	S	S	MDR	MSSA
SA-64	0.5	2	<0.5	S	S	S	S	R	R	S	S	S	S	R	MSSA
SA-65	1	2	<0.5	S	S	S	S	R	S	R	S	S	S	R	MSSA
SA-66	1	2	<0.5	S	S	S	S	R	S	S	S	S	S	R	MSSA
SA-67	1	4	0.25	S	S	S	S	S	S	R	S	R	R	MDR	MSSA
SA-68	1	4	0.5	S	S	S	S	R	S	R	S	S	R	MDR	MSSA
SA-69	0.5	4	8	S	S	R	R	R	S	S	S	S	R	MDR	MRSA
SA-70	1	4	8	S	S	R	R	S	R	R	S	S	R	MDR	MRSA
SA-71	1	4	32	S	S	R	R	R	R	R	S	S	S	MDR	MRSA
SA-72	2	4	32	S	S	R	R	R	R	R	S	S	R	MDR	MRSA
SA-73	2	4	0.5	S	S	S	S	S	S	R	S	S	R	R	MSSA
SA-74	1	4	8	S	S	R	R	R	S	R	S	S	R	MDR	MRSA
SA-75	2	4	0.5	S	S	R	R	S	S	S	S	S	S	R	MRSA
SA-76	1	4	0.5	S	S	R	R	R	S	R	S	S	R	MDR	MRSA
SA-77	2	4	32	S	S	R	R	R	R	R	S	S	R	MDR	MRSA
SA-78	1	4	2	S	S	S	S	R	R	R	S	S	R	MDR	MSSA
SA-79	1	2	16	S	S	R	R	S	R	R	S	S	R	MDR	MRSA
SA-80	2	2	0.5	S	S	R	R	S	R	R	R	R	S	MDR	MRSA

Sensitive (S), resistant (R), multidrug resistance (MDR), vancomycin (VAN), linezolid (LIN), oxacillin (OXA), cefoxitime (CX30), clindamycin (CD2), erythromycin (E15), gentamicin (GEN10), tetracycline (TE30), teicoplanin (TEI30) and rifampicin (RIF5).

**Table 3:** PCR based screening of antimicrobial resistance genes in MRSA isolates.

Isolate ID	*mecA*	*mecC*	*vanA*	SCC*mec* Type
SA-23	+	-	-	IVd
SA-24	+	-	-	II
SA-25	+	-	-	II
SA-27	-	-	-	
SA-28	+	-	-	II
SA-29	+	-	-	II
SA-38	+	-	-	III
SA-44	+	-	-	III
SA-46	+	-	-	III
SA-51	+	-	-	III
SA-69	+	-	-	III
SA-70	+	-	-	III
SA-71	+	-	-	III
SA-72	-	-	-	
SA-74	-	-	-	
SA-75	-	-	-	
SA-76	+	-	-	Iva
SA-77	+	-	-	Iva
SA-79	+	-	-	Iva
SA-80	+	-	-	Iva

(+) = presence of gene, (-) = absence of gene.

## Data Availability

Not applicable.
